# Changes in metabolic syndrome affect the health-related quality of life of community-dwelling adults

**DOI:** 10.1038/s41598-021-99767-y

**Published:** 2021-10-12

**Authors:** Yi-Hsuan Lin, Hsiao-Ting Chang, Yen-Han Tseng, Harn-Shen Chen, Shu-Chiung Chiang, Tzeng-Ji Chen, Shinn-Jang Hwang

**Affiliations:** 1 Department of Family Medicine, Cheng Hsin General Hospital, Taipei, Taiwan; 2 Faculty of Medicine, School of Medicine, National Yang Ming Chiao Tung University, Taipei, Taiwan; 3 Department of Family Medicine, Taipei Veterans General Hospital, Taipei, Taiwan; 4 Department of Chest Medicine, Taipei Veterans General Hospital, Taipei, Taiwan; 5 Division of Endocrinology and Metabolism, Department of Medicine, Taipei Veterans General Hospital, Taipei, Taiwan; 6 Institute of Hospital and Health Care Administration, School of Medicine, National Yang Ming Chiao Tung University, Taipei, Taiwan

**Keywords:** Endocrinology, Health care

## Abstract

Metabolic syndrome (MetS) is associated with cardiovascular diseases, type 2 diabetes, chronic renal diseases, and all-cause mortality. Furthermore, MetS is associated with poor health-related quality of life (HRQOL). However, the impact of dynamic changes in MetS on changes in the HRQOL was not previously explored. This was an eight-year, prospective cohort study in which 906 middle-aged adults from Shipai, Taipei in northern Taiwan were enrolled during 2009–2010 (baseline). Of those sampled, 427 participants completed the follow-up investigation after 8 years. The HRQOL was measured using the Short Form Health Survey (SF-36). Other variables including age, sex, marital status, level of education, smoking, alcohol consumption, baseline body mass index, and changes in physical activity were adjusted. Compared with adults who never experienced MetS, adults with persistent MetS had a negative change in mental HRQOL (β − 4.20, 95% CI − 7.54 to − 0.86, *p* = 0.01). The negative changes of persistent MetS on the HRQOL were in the domains of vitality and mental health (β − 4.42, 95% CI − 8.10 to − 0.73 and β − 3.47, 95% CI − 6.90 to − 0.04, respectively). Women and overweight adults were vulnerable to the detrimental effects of persistent MetS. For better HRQOL, more resources should be devoted to reversing MetS in public health.

## Introduction

Metabolic syndrome (MetS) is a constellation of metabolic disorders including abdominal obesity, hypertension, hyperglycemia and dyslipidemia^1^. Insulin resistance and chronic subclinical inflammatory status are essential factors in the pathogenesis of MetS^2,3^. MetS is associated with cardiovascular diseases, type 2 diabetes, chronic renal diseases and all-cause mortality^4–6^. MetS contributes substantially to non-communicable diseases that, result in approximately 41 million deaths yearly, worldwide^7^.

Beyond medical consequences, MetS is also associated with the health-related quality of life (HRQOL). The improvement of health services leads to an extension in lifespan; hence, researchers have focused on the quality of life. The HRQOL refers to the aspects of quality of life that relate to health^8^. It is an overall perception of an individual’s feeling of how well they function in their life, including physical, mental and social aspects^9^. Many tools have been used to evaluate the HRQOL. However, the Short Form Health Survey (SF-36) has reliability and validity^10^, and is the most widely used instrument^11^.

A growing number of studies have investigated the association between MetS and the HRQOL. Most studies have shown that MetS is associated with a lower HRQOL^12–14^. However, the results are diverse. A study from Japan found that a number of MetS components was negatively associated with general health but positively associated with mental health^15^. A study in Korea did not find a significant association between MetS and the HRQOL after adjusting for confounding factors^16^. Furthermore, sex and body mass index (BMI) may affect the relationship between MetS and the HRQOL. Some studies have observed the detrimental effects of MetS on the HRQOL only in women^17–19^. Another study in Korea showed that individuals with MetS suffered from a stronger impairment in obesity-specific quality of life^20^. Upon reviewing the past literature, we found that most studies on MetS and the HRQOL had cross-sectional designs. Changes in MetS and the HRQOL were not considered. Whether the effects of sex and BMI were significant when the changes in MetS and the HRQOL were analyzed, is also unknown.

Our study was a community-based, 8-year cohort study. We aimed to explore the association between changes in MetS and changes in the HRQOL. We further assessed whether sex and BMI modified the association. To the best of our knowledge, the relationship between changes in MetS and changes in the HRQOL has never been investigated. By demonstrating the effects of a change in MetS on the HRQOL, we provide more evidence on this key issue.

## Methods

### Study design and population

This prospective cohort study was approved by the Research Ethics Committee of Taipei Veterans General Hospital, Taipei, Taiwan on March 17, 2017 (Protocol Code: 201701-009BCF). All methods were performed in accordance with the relevant guidelines and regulations. During 2009–2010 (baseline), residents who lived in the Shilin and Beitou districts of Taipei, Taiwan for more than six months were candidates for the study population. Randomized sampling was conducted of residents aged 35–55 years. The residents who agreed to participate in the study signed an informed consent documents before recruitment. At baseline, 906 adults were enrolled in the study. The participants were followed up after eight years (2017–2018). A total of 427 participants completed the follow-up examination and were included in the analyses. The mean follow-up period was 8.6 ± 0.6 years. The baseline age of study population was between 35 to 55 years, with a median age of 48.1 years. 59.3% of the study participants were women.

### Diagnostic criteria of metabolic syndrome

MetS was evaluated at both the baseline and the eight-year follow-up investigation. The diagnostic criteria for MetS were based on the National Cholesterol Education Program Adult Treatment Panel III (ATP III) guidelines^1^. The components of MetS were (1) abdominal obesity: waist circumference ≥ 90 cm in men and ≥ 80 cm in women (for Asians); (2) hypertriglyceridemia: serum triglyceride ≥ 150 mg/dL, or on drug treatment; (3) low serum high-density lipoprotein cholesterol (HDL-C): HDL-C < 40 mg/dL for men or < 50 mg/dL for women, or on drug treatment (4) hypertension: blood pressure ≥ 130/85 mmHg, or on drug treatment; and (5) hyperglycemia: serum fasting glucose ≥ 100 mg/dL, or receiving drug treatment. Participants who satisfied three or more components were classified into the MetS group. According to a change in MetS status, participants could be grouped into four categories: never had MetS, improved MetS status (presence of MetS at baseline but absence at follow-up), progressed MetS status (absence of MetS at baseline but presence at follow-up), and persistent MetS groups.

### Measurement of health-related quality of life

We used the Taiwan version of SF-36^21^ to evaluate the HRQOL. The results of norming and validation for the SF-36 Taiwan version were similar to those of the United States version^22^. The SF-36 Taiwan version has been proven to have good discriminant validity and internal reliability^21^. The SF-36 includes eight domains: physical functioning, role participation with physical health problems (role-physical), bodily pain, general health, vitality, social functioning, role participation with emotional health problems (role-emotional), and mental health problems. According to the scoring procedures provided by the user's manual for the SF-36^23^, the eight domain scales could be calculated to attain the physical health component summary (PCS) and the mental health component summary (MCS) by different weighting and transformation. Higher scores represented a better HRQOL. The SF-36 questionnaire was administered at baseline and at the eight-year follow-up examination. The changes in the PCS, MCS, and each domain of SF-36 were calculated by subtracting the scores of the PCS, MCS, and each domain of SF-36 at baseline from the scores at the follow-up assessments.

### Covariates

Basic demographic data including age, sex, marital status, education, cigarette smoking, alcohol consumption, medical history, and medication use were self-reported. Cigarette smoking was categorized as non-smokers, smokers and ex-smokers (individuals who had stopped smoking). Medical history included hypertension, diabetes mellitus, and hyperlipidemia. Medication use included antihypertensive agents, oral hypoglycemic agents, insulin, statins and other lipid-lowering agents.

During the interviews at baseline and the follow-up assessment, blood pressure (mmHg), body weight (kg), body height (cm), and waist circumference (cm) were measured by well-trained research nurses. Blood pressure was measured for three times to obtain the average blood pressure for analyses. Blood tests were performed for each participant to obtain laboratory data including fasting glucose (mg/dL), serum HDL-C (mg/dL), and triglycerides (mg/dL).

Physical activity was evaluated using the International Physical Activity Questionnaire (IPAQ) Short-Form, Taiwan version^24^. Participants reported the duration and frequency of walking, and moderate, and high intensity activities in the previous week. Participants were categorized into high, moderate and low physical activity groups according to the scoring principle of the IPAQ^25^. In our main analyses, we corrected the change in physical activity to adjust the effects of changes in physical activity on changes in the HRQOL^26^. Participants who maintained their physical activity in moderate or high activity groups during the study period were categorized as the always active group. The became active group referred to those participants who had low physical activity at baseline and became moderately or highly physically active by the follow-up evaluation. The became inactive group included participants with moderate or high physical activity at baseline and low physical activity at the follow-up assessment. Participants who maintained low physical activity at baseline and the follow-up appraisal were classified as always inactive.

### Statistical analyses

For the descriptive analyses, we performed chi-square tests and Fisher's exact tests for categorical, and t-tests for continuous variables. Multiple linear regression models were used for the main analyses. We conducted stepwise procedures for fitting regression models. The final model adjusted for age, sex, marital status, level of education, cigarette smoking, alcohol consumption, baseline BMI, and change in physical activity. We investigated the associations between changes in MetS and changes in the PCS, MCS, and each SF-36 domain. Stratified analyses according to sex and baseline BMI were performed. A two-tailed *p* value of < 0.05 was considered statistically significant. SAS version 9.4 (SAS Institute Inc., Cary, NC, USA) was used for all statistical analyses.

## Results

### Demographic characteristics at baseline and the eight-year follow-up

Table 1 shows the demographic data of the study population at baseline. The distribution of age in participants was 35–55 years. About half of the participants were between 40 and 49 years, with a mean age of 47.2 ± 5.4 years. Women (253 participants) comprised 59.3% of the total sample. The prevalence of MetS at baseline was 14.8%. The proportion of women was higher in the non-MetS group than in the MetS group (63.5% vs. 34.9%). Participants with MetS at baseline were likely to be overweight than those without MetS (87.3% vs. 32.4%). Table 2 shows the characteristics of the participants at baseline and at the 8-year follow-up. After 8 years, at the follow-up assessment, the prevalence of low physical activity and MetS increased (35.4 to 51.5% for low physical activity and 14.8 to 25.1% for MetS). The HRQOL showed improvements in the MCS but deterioration in the PCS (mean PCS score as 55.0 ± 6.7 to 52.9 ± 6.2, and mean MCS score as 38.0 ± 4.9 to 48.7 ± 8.2). There were no significant differences in smoking, alcohol consumption, and BMI during the study period.

**Table 1:** Demographic characteristics of the study population at baseline (n = 427).

	No MetS	MetS	*p*
	n = 364 (85.2%)	n = 63 (14.8%)	
**Age in years, n (%)**			
30–39	46 (12.6)	8 (12.7)	0.44
40–49	191 (52.5)	28 (44.4)	
50–59	127 (34.9)	27 (42.9)	
Mean age (SD)	47.2 (5.3)	47.3 (5.8)	0.93
Women, n (%)	231 (63.5)	22 (34.9)	< 0.001
**Marital status**^**a**^**, n (%)**			
Married	313 (86.5)	53 (85.5)	0.84
Single/divorced/separated/widowed/others	49 (13.5)	9 (14.5)	
**Cigarette smoking**^**a**^**, n (%)**			
Non-smokers	295 (81.7)	50 (79.4)	0.87
Smokers	43 (11.9)	9 (14.3)	
Ex-smokers	23 (6.4)	4 (6.4)	
**Alcohol consumption**^**a**^**, n (%)**			
No	304 (83.8)	54 (85.7)	0.69
Yes	59 (16.3)	9 (14.3)	
**Physical activity**^**b**^**, n (%)**			
Low	129 (35.4)	22 (34.9)	0.57
Moderate	152 (41.8)	30 (47.6)	
High	83 (22.8)	11 (17.5)	
**Education**^**a**^**, n (%)**			
Illiterate/elementary school	8 (2.2)	1 (1.6)	0.62
Senior/junior high school	127 (35.0)	26 (41.3)	
University and above	228 (62.8)	36 (57.1)	
**BMI (kg/m**^**2**^**), n (%)**			
< 18.5	11 (3.0)	0	< 0.001
18.5–23.9	235 (64.6)	8 (12.7)	
≥ 24	118 (32.4)	55 (87.3)	
Mean BMI (SD)	23.1 (3.0)	27.7 (4.1)	< 0.001

Chi-square tests and Fisher’s exact tests were used for categorical variables.

T-tests were used for continuous variables.

*MetS* metabolic syndrome, *SD* standard deviation, *BMI* body mass index.

^a^There were few missing data in these covariates.

^b^Physical activity was evaluated by the International Physical Activity Questionnaire (IPAQ) Short-Form, Taiwan version.

**Table 2:** Characteristics of the study population at baseline and the eight-year follow-up (n = 427).

	Baseline	8-year follow-up	*p*
**Cigarette smoking**^**a**^**, n (%)**			
Non-smokers	345 (81.4)	361 (84.7)	0.15
Smokers	52 (12.3)	35 (8.2)	
Ex-smokers	27 (6.4)	30 (7.0)	
**Alcohol consumption**^**a**^**, n (%)**			
No	358 (84.0)	340 (79.8)	0.11
Yes	68 (16.0)	86 (20.2)	
**BMI (kg/m**^**2**^**), n (%)**			
< 18.5	11 (2.6)	17 (4.0)	0.47
18.5–23.9	243 (56.9)	234 (54.7)	
≥ 24	173 (40.5)	176 (41.2)	
Mean BMI (SD)	23.8 (3.6)	23.9 (4.0)	0.61
**Physical activity**^**b**^**, n (%)**			
Low	151 (35.4)	220 (51.5)	< 0.001
Moderate	182 (42.6)	149 (34.8)	
High	94 (22.0)	58 (13.6)	
**Prevalence of metabolic syndrome (MetS), n (%)**	63 (14.8)	107 (25.1)	< 0.001
**Number of MetS components, mean (SD)**	1.2 (1.2)	1.7 (1.4)	< 0.001
**Prevalence of MetS components, n (%)**			
Abdominal obesity	181 (42.4)	141 (33.0)	0.004
Hypertension	133 (31.2)	217 (50.8)	< 0.001
Hyperglycemia	68 (15.9)	147 (34.4)	< 0.001
Low HDL-C	60 (14.1)	114 (26.7)	< 0.001
Elevated triglycerides	91 (21.3)	82 (19.2)	0.82
**SF-36, mean (SD)**			
Physical functioning	53.2 (4.8)	53.0 (5.0)	0.58
Role-Physical	51.9 (8.1)	51.7 (8.6)	0.80
Bodily pain	53.1 (7.8)	53.3 (8.0)	0.64
General health	47.8 (9.0)	47.1 (8.3)	0.25
Vitality	48.1 (4.7)	54.0 (7.5)	< 0.001
Social functioning	31.6 (3.9)	50.3 (6.1)	< 0.001
Role-Emotional	50.4 (9.8)	50.6 (9.5)	0.77
Mental health	38.5 (4.1)	47.0 (8.2)	< 0.001
Physical Component Summary	55.0 (6.7)	52.9 (6.2)	< 0.001
Mental Component Summary	38.0 (4.9)	48.7 (8.2)	< 0.001

Chi-square tests were used for categorical variables.

T-tests were used for continuous variables.

*BMI* body mass index, *SD* standard deviation, *MetS* metabolic syndrome.

^a^There were few missing data in these covariates.

^b^Physical activity was evaluated by the International Physical Activity Questionnaire (IPAQ) Short-Form, Taiwan version.

Sex differences of demographic characteristics were shown in Supplementary Table S1. Women had lower ratios of being married (83.3% vs. 90.7%) and being highly educated (university and above: 55.9 vs. 70.7%) than men. Women were less likely to have habits of cigarette smoking (3.6% vs. 24.9%) and alcohol consumption (6.3% vs. 29.9%). Women also had a lower mean BMI than men at both baseline (22.7 kg/m^2^ vs. 25.3 kg/m^2^) and follow-up (22.8 kg/m^2^ vs. 25.5 kg/m^2^). During the study period, there were 298 (69.8%) participants who never had MetS. 22 (5.2%) participants had an improved MetS status. 66 (15.5%) individuals were categorized as progressed MetS group. 41 (9.6%) subjects had persistent MetS. Women had a higher ratio of never had MetS (77.9% vs. 58.1%), and lower ratios of progressed and persistent MetS status (13.4% vs. 18.4% and 5.9% vs. 14.9%, respectively). There were no significant sex differences in mean age at baseline, change in BMI, physical activity at baseline, and change in physical activity.

### The association between changes in MetS and changes in HRQOL

Table 3 shows the association between changes in MetS with changes in the PCS and MCS after adjusting for age, sex, baseline BMI, marital status, level of education, cigarette smoking, alcohol consumption, and changes in physical activity. Compared with participants who never had MetS, adults with a persistent positive MetS status had a negative change in the MCS (β − 4.20, 95% CI − 7.54 to − 0.86, *p* = 0.01). However, changes in MetS did not significantly affect changes in the PCS. Other protective factors for change in the MCS included higher levels of education, alcohol consumption, and being physically active. Table 4 shows the association between changes in MetS and changes in each domain of the SF-36. Compared with adults who did not have MetS, participants with a persistent MetS status performed worse in changes of vitality (β − 4.42, 95% CI − 8.10 to − 0.73, *p* = 0.02) and mental health (β − 3.47, 95% CI − 6.90 to − 0.04, *p* = 0.047).

**Table 3:** The association between change in metabolic syndrome and change in health-related quality of life (n = 427).

	Change in physical component summary		Change in mental component summary	
	β (95% CI)	*p*	β (95% CI)	*p*
**Change in metabolic syndrome (MetS)**^**a**^				
Never had MetS	Ref		Ref	
Improved	0.52 (− 2.77, 3.82)	0.76	− 1.70 (− 5.74, 2.34)	0.41
Progressed	1.30 (− 0.80, 3.39)	0.22	− 2.07 (− 4.63, 0.49)	0.11
Persistent MetS	2.01 (− 0.72, 4.73)	0.15	− 4.20 (− 7.54, − 0.86)	0.01
Age	0.03 (− 0.10, 0.16)	0.64	− 0.01 (− 0.17, 0.16)	0.94
Women	− 1.01 (− 2.73, 0.72)	0.25	1.58 (− 0.53, 3.70)	0.14
Baseline BMI (kg/m^2^)	− 0.22 (− 0.47, 0.03)	0.08	0.14 (− 0.17, 0.45)	0.37
**Marital status**				
Single/divorced/separated/widowed/others	Ref		Ref	
Married	− 0.24 (− 2.32, 1.83)	0.82	0.37 (− 2.17, 2.92)	0.77
**Education**				
Illiterate/elementary school	Ref		Ref	
Senior/junior high school	− 0.55 (− 5.80, 4.70)	0.84	6.99 (0.55, 13.43)	0.03
University and above	− 1.61 (− 6.88, 3.66)	0.55	7.69 (1.23, 14.16)	0.02
**Cigarette smoking**				
Non-smokers	Ref		Ref	
Smokers	− 0.01 (− 2.33, 2.31)	0.99	0.97 (− 1.87, 3.82)	0.50
Ex-smokers	0.59 (− 2.57, 3.76)	0.71	0.69 (− 3.18, 4.57)	0.73
Alcohol	− 1.83 (− 3.94, 0.28)	0.09	2.69 (0.10, 5.28)	0.04
**Change in physical activity**^**b**^				
Always inactive	Ref		Ref	
Became inactive	− 0.17 (− 2.15, 1.81)	0.87	2.26 (− 0.17, 4.70)	0.07
Became active	1.59 (− 0.76, 3.94)	0.18	0.92 (− 1.96, 3.80)	0.53
Always active	− 0.46 (− 2.42, 1.50)	0.65	2.84 (0.43, 5.25)	0.02

The analyses were conducted by multivariable linear regression.

*MetS* metabolic syndrome, *BMI* body mass index.

^a^There were 298 (69.8%) participants who never had MetS. 22 (5.2%) participants had an improved MetS status. 66 (15.5%) individuals were categorized as progressed MetS group. 41 (9.6%) subjects had persistent MetS.

^b^Physical activity was evaluated by the International Physical Activity Questionnaire (IPAQ) Short-Form, Taiwan version. Participants who had low physical activity at baseline and became moderately or highly physically active at follow-up were categorized as the became active group. Participants who had moderate or high physical activity at baseline and low physical activity at follow-up were categorized as the became inactive group.

**Table 4:** The association between change in metabolic syndrome and change in SF-36 domains (n = 427).

Change in the domains of SF-36	Never had MetS^a^	Improved^a^		Progressed^a^		Persistent MetS^a^	
		β (95% CI)	*p*	β (95% CI)	*p*	β (95% CI)	*p*
Physical functioning	Ref	0.51 (− 2.00, 3.01)	0.69	1.42 (− 0.17, 3.01)	0.08	0.37 (− 1.74, 2.48)	0.73
Role-physical	Ref	− 2.33 (− 6.73, 2.08)	0.30	0.86 (− 1.92, 3.63)	0.55	− 0.72 (− 4.44, 2.99)	0.70
Bodily pain	Ref	1.06 (− 3.02, 5.13)	0.61	− 0.48 (− 3.05, 2.08)	0.71	2.53 (− 0.91, 5.96)	0.15
General health	Ref	2.41 (− 1.30, 6.12)	0.20	1.03 (− 1.32, 3.37)	0.39	1.52 (− 1.61, 4.64)	0.34
Vitality	Ref	− 1.86 (− 6.21, 2.50)	0.40	− 1.54 (− 4.27, 1.19)	0.27	− 4.42 (− 8.10, − 0.73)	0.02
Social functioning	Ref	0.21 (− 2.79, 3.21)	0.89	− 1.04 (− 2.94, 0.85)	0.28	− 0.41 (− 2.93, 2.12)	0.75
Role-emotional	Ref	− 4.31 (− 9.33, 0.71)	0.09	− 1.50 (− 4.67, 1.67)	0.35	− 2.77 (− 7.00, 1.46)	0.20
Mental health	Ref	0.43 (− 3.72, 4.58)	0.84	− 0.37 (− 2.95, 2.21)	0.78	− 3.47 (− 6.90, − 0.04)	0.047

Multivariable linear regression models were adjusted for age, sex, marital status, level of education, smoking, alcohol consumption, baseline body mass index, and change in physical activity.

*SF-36* the Short Form Health Survey, *MetS* metabolic syndrome.

^a^There were 298 (69.8%) participants who never had MetS. 22 (5.2%) participants had an improved MetS status. 66 (15.5%) individuals were categorized as progressed MetS group. 41 (9.6%) subjects had persistent MetS.

### Stratified analyses by sex and baseline BMI

Compared with male participants who did not have MetS, men with a progressed MetS status had a negative change in their MCS (β − 4.23, 95% CI − 7.90 to − 0.56, *p* = 0.02, Table 5). Female participants with a persistent MetS status had an average change in their MCS of − 6.68 (95% CI − 11.82 to − 1.54, *p* = 0.01) compared to those women without MetS. Among participants who were overweight at baseline, individuals with a persistent MetS status had a negative change in their MCS compared with those who did not have MetS (β − 4.03, 95% CI − 7.43 to − 0.63, *p* = 0.02). For participants who were not overweight, we did not observe a significant impact of changes in MetS on changes in HRQOL.

**Table 5:** The associations of change in metabolic syndrome and change in health-related quality of life, stratified by sex and baseline BMI (n = 427).

	Change in PCS		Change in MCS		Change in PCS		Change in MCS	
	β (95% CI)	*p*	β (95% CI)	*p*	β (95% CI)	*P*	β (95% CI)	*p*
**Model 1**	**Men** (n = 174)				**Women** (n = 253)			
**Change in MetS**^**a**^								
Never had MetS	Ref		Ref		Ref		Ref	
Improved	− 0.62 (− 4.16, 2.92)	0.73	− 0.86 (− 5.70, 3.97)	0.73	2.52 (− 3.23, 8.27)	0.39	− 3.29 (− 10.07, 3.49)	0.34
Progressed	1.64 (− 1.04, 4.33)	0.23	− 4.23 (− 7.90, − 0.56)	0.02	0.86 (− 2.10, 3.82)	0.57	0.22 (− 3.27, 3.71)	0.90
Persistent MetS	1.42 (− 1.71, 4.55)	0.37	− 2.73 (− 7.01, 1.55)	0.21	3.48 (− 0.88, 7.84)	0.12	− 6.68 (− 11.82, − 1.54)	0.01
**Model 2**	**Baseline BMI < 24** (n = 254)				**Baseline BMI** ≥ **24** (n = 173)			
**Change in MetS**^**a**^								
Never had MetS	Ref		Ref		Ref		Ref	
Improved	0.68 (− 4.73, 6.09)	0.81	− 2.09 (− 8.79, 4.61)	0.54	0.30 (− 3.89, 4.50)	0.89	− 0.80 (− 5.87, 4.27)	0.76
Progressed	− 0.12 (− 3.29, 3.05)	0.94	− 2.91 (− 6.83, 1.01)	0.15	1.61 (− 1.18, 4.41)	0.26	− 1.19 (− 4.57, 2.19)	0.49
Persistent MetS	2.88 (− 10.48, 16.25)	0.67	7.27 (− 9.28, 23.82)	0.39	1.55 (− 1.27, 4.36)	0.28	− 4.03 (− 7.43, − 0.63)	0.02

Model 1 was adjusted for age, marital status, level of education, smoking, alcohol consumption, baseline BMI, and change in physical activity.

Model 2 was adjusted for age, sex, marital status, level of education, smoking, alcohol consumption, and change in physical activity.

*MetS* metabolic syndrome, *PCS* physical component summary, *MCS* mental component summary, *BMI* body mass index.

^a^There were 298 (69.8%) participants who never had MetS. 22 (5.2%) participants had an improved MetS status. 66 (15.5%) individuals were categorized as progressed MetS group. 41 (9.6%) subjects had persistent MetS.

## Discussion

We found that a persistent MetS status predicted a deterioration in mental HRQOL among community-dwelling middle-aged adults, especially in the domains of vitality and mental health. Men with a progressive MetS status, and women and overweight adults with a persistent MetS status, were vulnerable to impaired mental HRQOL.

An epidemiological study of MetS in Taiwan showed that the prevalence of MetS ranged from 10.7 to 23% in adults aged 40–49 years old^27^, which was consistent with the prevalence of MetS in this study (14.8%). Saboya et al. reviewed studies on the association between MetS and quality of life. Most studies have concluded that MetS is significantly related to a poor quality of life^28^. However, almost all studies discussing MetS and quality of life were cross-sectional and causal inferences could not be established. A 7-year cohort study reported that MetS was a risk factor for impaired quality of life among older adults^29^, while MetS was only assessed at baseline. Our study found that a persistent MetS status had harmful effects on the change in mental HRQOL, especially in vitality and mental health. This result is slightly different from that of previous studies. Hjellset et al. reported that MetS was associated with lower general health, physical functioning, and more bodily pain among Pakistani immigrant women^30^. Even with a study population of similar age group and the same tool (SF-36) to evaluate the HRQOL as in our study, Frisman et al. reported that people with MetS had lower scores on the physical and social dimensions of SF-36^31^. Ethnic, cultural and environmental factors may modify the association between MetS and the HRQOL.

MetS was found to be associated with depressive symptoms, poorer self-rated health, and low social support^32–34^. The possible link between MetS and poor mental health may be obesity and multiple chronic illnesses. Obesity and low levels of leptin are associated with a depressed mood^35^. The symptoms and complications of chronic illness and related treatments also cause poor mental HRQOL^36^. For example, the duration of disease and the level of fasting blood sugar were predictors of impaired HRQOL among patients with type II diabetes^37^. Psychiatric disorders, including depression and anxiety, were found to be common among diabetic patients, and diabetic patients with psychiatric disorders were more vulnerable to impaired HRQOL^38^. Our study showed that a persistent MetS status resulted in a deteriorated change in mental HRQOL. On the other hand, neither physical nor mental HRQOL was significantly affected if a MetS status was not persistent. That is, the harmful effect of a persistent MetS status on the HRQOL could be reversed by the improvement of MetS. This inference is in line with a previous randomized control trial. Kalter-Leibovici et al. reported that intensive lifestyle interventions decreased the prevalence of MetS and improved the HRQOL^39^.

In our results, women were less likely to suffer from MetS and could maintain the MetS free status throughout the study period. It might be because the study participants were middle-aged adults. For women before menopause, estrogen plays a protective role in metabolic health, including increased insulin sensitivity, decreased central fat deposition, and improvement in lipid profile^40^. The result was also compactable with the past epidemiology study in Taiwan, which showed that women before menopause had a lower prevalence of MetS than men^27^.

Although women were less likely to have MetS, women were more vulnerable to the harmful effects of persistent MetS on mental HRQOL. The sex differences in the sociological background might explain the results. Compared with the male participants, a higher ratio of female participants in our study had a low level of education and a marital status of being single, divorced, separated, and widowed. A past study reported married people had better mental health and HRQOL than people with marriage problems^41^. In addition, higher levels of education showed a protective effect on the changes in the MCS in our results. Gil-Lacruz et al. also reported that a higher level of education was related to a better HRQOL, especially regarding the mental health dimension and in women^42^.

Our study found that being overweight and having a persistent MetS status had an interactive effect on mental HRQOL. Being overweight and dissatisfied with one’s body image were related to negative psychological well-being^43^. Nevertheless, our results showed that overweight adults with an improved MetS status did not demonstrate deterioration in the PCS and MCS compared to overweight adults who never had MetS. This result was consistent with Lopez-Garcia's research on Caucasians, which reported no significant impairment of the PCS and MCS of the HRQOL for people with metabolically healthy overweight^44^. However, previous studies about the interactive effects of metabolic health, HROQL, and obesity are rare and Asian data is lacking. Further research including different study populations is needed to investigate this important issue.

This study had some limitations. The participants of this study were Taiwanese adults so the generalizability may be limited. Information on income and family support were not available. Hence, we could only use the level of education and marital status to represent social standing and family support. Comorbidity, which may be a possible risk factor of a poorer HRQOL, was not adjusted in the model. However, adults with MetS tend to have multiple comorbidities. Comorbidity is likely to be a mediator rather than a confounder. In addition, there were some missing covariates in our analytical data. However, the proportion of missing data for these variables was so tiny (ranged from 0.2 to 0.7%) that the effect size of the main analysis was not affected. Finally, attrition bias may have occurred in this cohort study. Because participants who did not attend the follow-up assessment tended to have poorer health, attrition bias was likely to cause an underestimation of the main effects.

Despite its limitations, this was an 8-year cohort study, and we considered changes in MetS and the HRQOL through effective analyses. We also adjusted for the effects of changes in physical activity. Additionally, the study population was a representative sample that was randomly sampled from community-dwelling middle-aged adults. Furthermore, the HRQOL was effectively measured with the SF-36; a tool widely examined for its validity and reliability.

## Conclusions

This study found that persistent MetS was a risk factor of negative changes in mental HRQOL among community-dwelling middle-aged adults. Hence, for better HRQOL, the public health policy should invest more effort into strategies to reverse MetS. Further research is needed to clarify the possible interactive effects of obesity, MetS, and the HRQOL, especially for the relationship between metabolic health and the HRQOL among overweight and obese people of different ethnicities.

## Supplementary Information

Supplementary Information.

## Data Availability

The datasets are available from the corresponding author based on reasonable request.
