# Supplementary material for: Changes in metabolic syndrome affect the health-related quality of life of community-dwelling adults
Source: Sci Rep. 2021 Oct 12;11:20267. doi: 10.1038/s41598-021-99767-y (PMC8511017; doi:10.1038/s41598-021-99767-y)
Supplement: Supplementary file 1 — Supplementary Information. [file 41598_2021_99767_MOESM1_ESM.pdf]

Changes in metabolic syndrome affect the health-related quality of life of community-dwelling adults

Yi-Hsuan Lin[1,2], Hsiao-Ting Chang[2,3,*], Yen-Han Tseng[2,4], Harn-Shen Chen[2,5], Shu-Chiung Chiang[6], Tzeng-Ji Chen[2,3,6], Shinn-Jang Hwang[2,3]

1 Department of Family Medicine, Cheng Hsin General Hospital, Taipei, Taiwan

2 Faculty of Medicine, School of Medicine, National Yang Ming Chiao Tung University, Taipei, Taiwan

3 Department of Family Medicine, Taipei Veterans General Hospital, Taipei, Taiwan

4 Department of Chest Medicine, Taipei Veterans General Hospital, Taipei, Taiwan

5 Division of Endocrinology and Metabolism, Department of Medicine, Taipei Veterans General Hospital

6 Institute of Hospital and Health Care Administration, School of Medicine, National Yang Ming Chiao Tung University, Taipei, Taiwan

* Address correspondence to Hsiao-Ting Chang, MD, PhD, Department of Family Medicine, Taipei Veterans General Hospital, Taiwan.

E-mail: htchang.tw@gmail.com

Supplementary Table S1. Sex differences of demographic characteristics for the study population (n=427).

	**Men**	**Women**	***P***
	**n=174 (40.7%)**	**n=253 (59.3%)**	
Baseline age, mean (SD)	47.0 (0.4)	47.4 (0.3)	0.42
Marital status[a], n (%)			
Married	156 (90.7)	210 (83.3)	0.03
Single/divorced/separated/widowed/others	16 (9.3)	42 (16.7)	
Education[a], n (%)			
Illiterate/elementary school	1 (0.6)	8 (3.2)	0.003
Senior/junior high school	50 (28.7)	103 (40.9)	
University and above	123 (70.7)	141 (55.9)	
Cigarette smoking at baseline[a], n (%)			
Non-smokers	109 (63.0)	236 (94.0)	<0.001
Smokers	43 (24.9)	9 (3.6)	
Ex-smokers	21 (12.1)	6 (2.4)	
Alcohol consumption at baseline[a], n (%)			
No	122 (70.1)	236 (93.7)	<0.001
Yes	52 (29.9)	16 (6.3)	
BMI at baseline (kg/m^2), mean (SD)	25.3 (3.4)	22.7 (3.3)	<0.001
BMI at follow-up (kg/m^2), mean (SD)	25.5 (4.0)	22.8 (3.7)	<0.001
Change in BMI (kg/m^2), mean (SD)	0.15 (1.75)	0.12 (1.63)	0.88
Physical activity at baseline[b], n (%)			
Low	59 (33.9)	92 (36.4)	0.40
Moderate	71 (40.8)	111 (43.9)	
High	44 (25.3)	50 (19.8)	
Change in physical activity, n (%)			
Always inactive, n=88 (20.6%)	36 (20.7)	52 (20.6)	0.78
Became inactive, n=132 (30.9%)	52 (29.9)	80 (31.6)	
Became active, n=63 (14.8%)	23 (13.2)	40 (15.8)	
Always active, n=144 (33.7)	63 (36.2)	81 (32.0)	
MetS at baseline, n (%)	41 (23.6)	22 (8.7)	<0.001
Change in MetS, n (%)			
Never had MetS, n=298 (69.8%)	101 (58.1)	197 (77.9)	<0.001
Improved, n=22 (5.2%)	15 (8.6)	7 (2.8)	
Progressed, n=66 (15.5%)	32 (18.4)	34 (13.4)	
Persistent MetS, n=41 (9.6%)	26 (14.9)	15 (5.9)	

Abbreviations: SD, standard deviation; BMI, body mass index; MetS, metabolic syndrome.
[a] There were few missing data in these covariates.
[b] Physical activity was evaluated by the International Physical Activity Questionnaire (IPAQ) Short-Form, Taiwan version.
Chi-square tests and Fisher's exact tests were used for categorical variables.
T-tests were used for continuous variables.
